# Supplementary material for: A Critical View of the Application of the APEX Software (Aqueous Photochemistry of Environmentally-Occurring Xenobiotics) to Predict Photoreaction Kinetics in Surface Freshwaters
Source: Molecules. 2019 Dec 18;25(1):9. doi: 10.3390/molecules25010009 (PMC7017383; doi:10.3390/molecules25010009)
Supplement: Supplementary file 1 [file molecules-25-00009-s001.zip › 3.2.4_gcc-4.4.0.zip!/3.2.4_gcc-4.4.0/3.2.4_gcc-4.4.0/doc/octave/html/interpreter/C_002dStyle-I_002fO-Functions.html]

C-Style I/O Functions - Untitled


Previous: Basic Input and Output,
Up: Input and Output


---

### 14.2 C-Style I/O Functions

Octave's C-style input and output functions provide most of the
functionality of the C programming language's standard I/O library. The
argument lists for some of the input functions are slightly different,
however, because Octave has no way of passing arguments by reference.

In the following, file refers to a file name and `fid` refers
to an integer file number, as returned by `fopen`.

There are three files that are always available. Although these files
can be accessed using their corresponding numeric file ids, you should
always use the symbolic names given in the table below, since it will
make your programs easier to understand.

— Built-in Function: **stdin** ()  
> Return the numeric value corresponding to the standard input stream.
> When Octave is used interactively, this is filtered through the command
> line editing functions.
>
>
>
> **See also:** stdout, stderr.

— Built-in Function: **stdout** ()  
> Return the numeric value corresponding to the standard output stream.
> Data written to the standard output is normally filtered through the pager.
>
>
>
> **See also:** stdin, stderr.

— Built-in Function: **stderr** ()  
> Return the numeric value corresponding to the standard error stream.
> Even if paging is turned on, the standard error is not sent to the
> pager. It is useful for error messages and prompts.
>
>
>
> **See also:** stdin, stdout.

- Opening and Closing Files- Simple Output- Line-Oriented Input- Formatted Output- Output Conversion for Matrices- Output Conversion Syntax- Table of Output Conversions- Integer Conversions- Floating-Point Conversions- Other Output Conversions- Formatted Input- Input Conversion Syntax- Table of Input Conversions- Numeric Input Conversions- String Input Conversions- Binary I/O- Temporary Files- EOF and Errors- File Positioning
